# Practical Manual of Diseases of the Skin

**Published:** 1892-06

**Authors:** 


### BOOK NOTICES.

A Practical Manual of Diseases of the Skin. By George H. Rohé, M. D., Professor of Materia Medica, Therapeutics, and Hygiene, and formerly Professor of Dermatology in the College of Physicians and Surgeons, Baltimore, etc., etc. Assisted by J. Williams Lord, A. B., M. D., Lecturer on Dermatology and Bacteriology in the College of Physicians and Surgeons; Assistant Physician to the Skin Department in the Dispensary of Johns Hopkins Hospital. Philadelphia (1231 Filbert Street), and London. The F. A. Davis Co., Publishers. 1892. Price, $1.25, net.

This is a beautiful little volume of some three hundred and ten pages on diseases of the skin by two distinguished authors. It constitutes No. 13 in *The Physician's and Student's Ready-Reference Series*. It has not been written, so the authors tell us, with the understanding to supplant any of the numerous works for the dermatological specialist. Therefore little space is given to theoretical speculations upon pathology and etiology. However, the medical student and busy practitioner will find in its pages many points of value and interest. The authors have tried to give brief and exact descriptions of the various diseases considered, and to indicate the simplest and most direct methods of treatment. The needs of the practitioner have been continually kept in view. Theoretical questions have throughout the book been subordinated to matters of fact.

As a matter of course, from a homœopathic standpoint, we can't indorse the author's theories and practices. We consider diseases of the skin constitutionally. To treat diseases of the skin as merely local troubles concerning the skin only, as is mostly done by medical men of all schools, is in our humble opinion utter folly, yes, a crime against humanity.

The medical profession talks very learnedly about pathology and pathological anatomy.

There is no end of percussing and auscultating the human body. They hear the faintest murmurs of the chest. They detect râles of all descriptions —crepitant and subcrepitant, moist and bronchial, mucous and sibilant, sonorous and cavernous râles. But how about *curing?* Take for instance consumption. We are told that bacilli cause the disease. But what was it that caused the bacilli? What was in the system before bacilliary life became possible?

No, no, my allopathic friend, you are mistaken as regards curing diseases of the skin. And this is also intended for the would-be homœopathist. Read Hahnemann's *Organon* and be convinced that the trouble is constitutional, being fed from within, having its life from within, therefore it must be treated medicinally from within, and lay aside lotions and ointments as useless and harmful. W. S.

